# Effect of C‐Reactive Protein-to-Albumin Ratio on Prognosis in Gastric Cancer Patients

**DOI:** 10.7759/cureus.23972

**Published:** 2022-04-09

**Authors:** Ertugrul G Alkurt, Dogukan Durak, Veysel Barış Turhan, Ibrahim Tayfun Sahiner

**Affiliations:** 1 General Surgery, Hitit University Corum Erol Olcok Training and Research Hospital, Corum, TUR

**Keywords:** cancer, oncology, prognosis, biomarkers, gastric cancer

## Abstract

Purpose: The ratio of c-reactive protein-to-albumin (CRP/Alb) is a biochemical marker of systemic inflammatory response and has been associated with poor survival in cancer. The purpose of this study was to investigate the effect of CRP/Alb ratios on prognosis in gastric cancers.

Methods: This study included a retrospective review of a total of 147 patients with locally advanced gastric cancer. Mean platelet volume (MPV) was analyzed statistically to find a prognostic relationship between monocyte/lymphocyte ratio, platelet distribution volume (PDW), MPV/platelet, c-reactive protein/albumin ratio (CAR), and gastric cancer. Patients were staged according to the American Joint Cancer Committee (AJCC) Staging Guidelines.

Results: The CRP/Alb ratio was independently associated with overall survival (OS) in patients with gastric cancer (GC). The CAR was above 0.25 in 52.7% (77) of the patients and below 0.25 in 47.3% (69) of the patients. Patients under 0.25 had statistically longer survival rates.

Conclusion: A high preoperative CAR value could predict poor prognosis in locally advanced gastric patients. The same predictive value was not observed in other hematological parameters. This simple and cost-effective ratio can be used as a clinically accessible biomarker to assist clinicians in determining future treatment plans.

## Introduction

Gastric cancer (GC) is the second most prevalent cause of mortality among gastrointestinal malignancies and one of the most common malignant tumors globally [1]. Despite advancements in surgery and adjuvant treatment in recent decades, GC patients' survival prospects remain dismal [2].

In the early stages of gastric cancer, tumor markers such as carcinoembryonic antigen (CEA), alpha-fetoprotein (AFP), cancer antigen (CA) 19-9, CA 125, and CA 24-2 have been used for diagnosis and prognosis of the disease. However, none of these tumor markers is a definitive indicator for the diagnosis and prognosis of gastric cancer, therefore, it bears great importance to determine promising prognostic factors. Many hematological parameters have been studied to be used in the diagnosis and prognosis of different cancer types [3-7]. In pancreatic cancer patients, the platelet/lymphocyte ratio has been recommended as a major prognostic predictor [8]. In hepatocellular carcinoma, the c-reactive protein/albumin ratio (CAR) has been described as an independent predictive factor [9]. Mean platelet volume (MPV), thrombocytosis, and CAR have been investigated separately as prognostic factors in patients with gastric cancer. Therefore, the aim of this study was to compare the prognostic value of systemic inflammatory markers in patients undergoing gastric cancer surgery.

## Materials and methods

Study population

Patients who were diagnosed and operated on in the general surgery clinic of Hitit University between May 2015 and October 2021 were retrospectively screened after receiving consent from the local ethics committee (Hitit University Non-Interventional Research Ethics Committee, decision no: 2021-81). A total of 147 cases of locally advanced gastric carcinoma were included in the study. Demographic data (age, gender), length of hospitalization, and preoperative hematological parameters MPV, monocyte, lymphocyte, platelet distribution volume (PDW), c-reactive protein (CRP), and albumin of all patients were obtained from digital records. In addition, the number of removed lymph nodes, the number of metastatic lymph nodes, tumor size, tumor type, and survey status were recorded. A history of blood transfusion within the previous two months, active bleeding, bleeding diathesis, hyper- or hypothyroidism, infections, disseminated intravascular coagulation, heparin medication, or connective tissue disease were all considered exclusion criteria.

Patients admitted to the general surgery outpatient clinic had their venous blood drawn into tubes containing ethylene diamine tetraacetic acid (EDTA). A hemocytometer was used to analyze the blood. Preoperative blood analysis findings were analyzed retrospectively. The American Joint Cancer Committee (AJCC) recommendations were used to stage the patients. Patient charts and digital records were used to obtain survival statistics.

Diagnosis of gastric cancer and synchronous tumors was confirmed by endoscopy and endoscopic biopsy. Thoracic and abdominal tomography was routinely performed on all patients to determine whether distant metastases were present.

MPV was obtained from the patient's routine preoperative blood analysis. Calculated values were divided into two categories - low (<10.51) and high (≥10.51) [10]. The lymphocyte/monocyte ratio (LMR) was determined by dividing the number of lymphocytes by the number of monocytes. The calculated values were separated into two groups - 4.8 and >4.8 [11]. PDW was obtained from the patient's routine preoperative blood analysis. Calculated values were divided into two categories - low (< 11.95) and high (≥11.95) [12]. CAR was calculated by dividing the value of c-reactive proteins by albumin (g/L). Calculated values were divided into two categories - <0.25 versus ≥0.25 [13].

Statistical analysis

Frequency, percentage, arithmetic mean, standard deviation, 95% confidence intervals, and mean values were used to describe descriptive statistics. The Kolmogorov-Smirnov test was used to determine if continuous variables conformed to the normal distribution. The Kaplan-Meier test was used to do the survival analysis. The time from diagnosis until the patient's death or last known contact was referred to as overall survival (OS). Because the available patient data allowed for the computation of 12-month survival rates, the overall survival of the patients was determined over a one-year period. The log-rank test was used to compare subgroup survival rates. Statistical significance was defined as a p-value of less than or equal to 0.05.

## Results

The mean age of 146 patients included in the study was 68.9±11.1 years; 36 of the patients were female (24.6%) and 110 were male (75.4%). Patients' preoperative serological and hematological data are summarized in Table 1.

**Table 1:** Patients’ hematological values before treatment MPV: mean platelet volume; LMR: lymphocyte/monocyte ratio; PDW: thrombocyte distribution width; CAR: CRP/albumin ratio; CRP: c-reactive protein

Variables	Median	Range
Age, years	68.5	36-95
MPV	9.8	7.8-13
LMR	3.04	0.51-20.6
PDW	11.7	8.1-18.4
CAR	0.21	0.06-3.45

**Figure 1:**
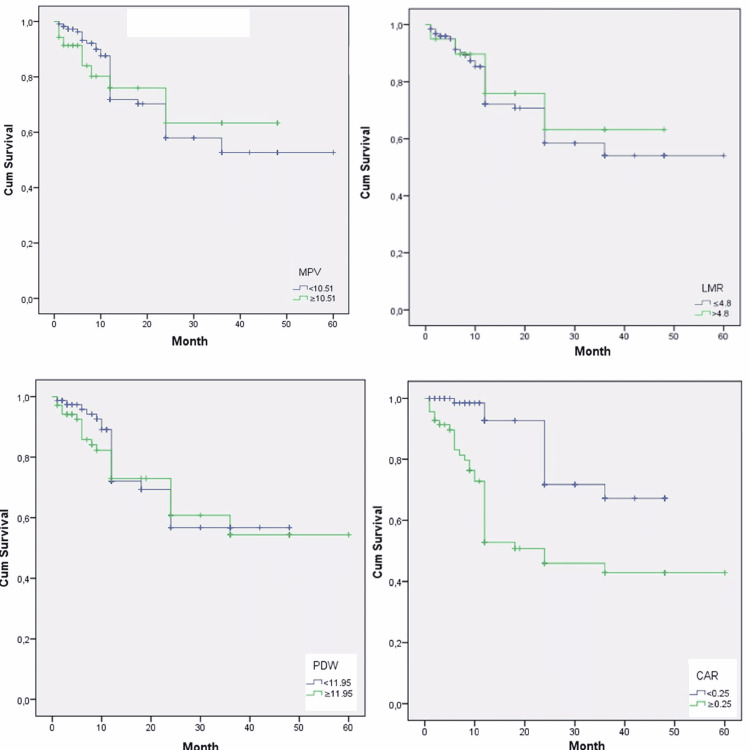
Survival curves for locally advanced gastric cancer patients stratified by MPV, LMR, PDW, and CAR MPV: mean platelet volume; LMR: lymphocyte/monocyte ratio; PDW: thrombocyte distribution width; CAR: CRP/albumin ratio; CRP: c-reactive protein

There was no statistical difference between patients with an MPV less than 10.51 (n = 111, 76.02%) and patients with an MPV greater than or equal to 10.51 (n = 35, 23.98%) in terms of median OS time (12 months {95% CI: 15.5-20.5}, 12 months {95% CI: 11.7-22.2}, respectively; p = 0.805). There was no statistical difference between patients with an LMR less than or equal to 4.8 (n = 126, 86.3%) and patients with LMR greater than 4.8 (n = 20, 23.7%) in terms of median OS time (12 months {95% CI: 15.4-20.3}, 12 months {95% CI: 11.3-23.4}, respectively; p = 0.646) (Figure 1).

There was no statistical difference between patients with a PDW less than 11.95 (n = 77, 52.7%) and patients with a PDW greater than or equal to 11.95 (n = 69, 47.3%) in terms of median OS time (12 months {95% CI: 13.2-18.6}, 12 months {95% CI: 16-23.6}, respectively; p = 0.761) (Table 2). A total of 77 (52.7%) patients had a CAR above 0.25. The CAR was above 0.25 in 52.7% (77) of the patients and below 0.25 in 47.3% (69) of the patients. Patients under 0.25 had statistically longer survival rates. The median survival time of patients below 0.25 was determined as 12 months (95% CI: 4-21.5), while those above 0.25 exhibited a median survival time of 12 months (95% CI: 13.5-20.4) (p<0.001).

**Table 2:** Case processing summary of the patient MPV: mean platelet volume; LMR: lymphocyte/monocyte ratio; PDW: thrombocyte distribution width; CAR: CRP/albumin ratio; CRP: c-reactive protein

Summary	Total (n)	Exitus (n, %)	Alive (n, %)
MPV			
<10.51	111	35, 31.5%	76, 68.5%
≥10.51	35	9, 25.7%	26, 74.3%
LMR			
≤4.8	126	39, 31%	87, 69.0%
>4.8	20	5, 25%	15, 75.0%
PDW			
-	77	21, 27.3%	56, 72.7%
≥11.95	69	23, 33.3%	46, 66.7%
CAR			
<0.25	77	12, 15.6%	65, 84.4%
≥0.25	69	32, 46.4%	37, 53.6%
Overall	146	44, 30.1%	102, 69.9%

## Discussion

In our study, we investigated 147 patients and found that the CAR ratio was a novel prognostic marker of locally advanced gastric cancers, but no significant difference was found among the other prognostic indices. Many studies have investigated the systemic inflammatory response which plays an important role in tumor progression and carcinogenesis in cancer patients [14-16]. The release of cytokines following tumor development as well as a microenvironment modulated by inflammatory cells stands as potent tumor promoters. Although this reaction against rapidly proliferating cells can be considered an antitumoral response, inflammatory responses are often deceptive in cancer patients [17].

The underlying mechanism has not been fully elucidated, but prognostic inflammatory values continue to be investigated. In reaction to IL-6, liver cells create serum CRP. Inflammation marks have been discovered to be a highly sensitive prognostic marker in a variety of primary malignancies, with low albumin in these patients [18,19]. Crumley et al. reported that low albumin exerts a negative effect on prognosis [20]. Similarly, Borda et al. concluded that preoperative treatment of hypoalbuminemia will positively contribute to the prognosis in patients with colorectal cancer [21]. Asher et al. found that albumin may be a predictor of survival in patients with ovarian cancer [22]. As a result, we might hypothesize that the CRP/Alb ratio (CAR), which is based on both high blood CRP and hypoalbuminemia, might be a stronger predictor of malignancy. At present, CAR is known to exhibit superior prognostic ability in ovarian, pancreatic, and small cell lung cancers compared to other prognostic indices [23-25]. Based on the cut-off value used by Lui et al., our study results also support that CAR is a strong prognostic marker for predicting the prognosis of GC patients [13].

Platelet size heterogeneity is measured by PDW. Platelet production rises when megakaryocytic maturation occurs, resulting in an increase in cytokines such as interleukin-6 (IL-6), granulocyte colony-stimulating factor (G-CSF), and macrophage colony-stimulating factor (MCSF) [12]. IL-6 is known to increase tumor angiogenesis and metastasis [26]. Tumor-activated platelets create a procoagulant microenvironment, allowing tumor cells to elude the immune system's detection. In the light of these data, many studies have previously been conducted to examine the relationship between PDW and cancer prognosis. It has been shown that PDW above a certain cut-off value indicates a poor prognosis in breast cancer, thyroid cancer, and colorectal cancer [27-29]. In our study, no significant relationship was found between PDW and gastric cancer prognosis. This may be because we used the cut-off value reported by Cheng et al. for gastric cancer [12].

Although recent studies have shown that an increased lymphocyte-monocyte ratio positively increases survival in gastrointestinal cancers, the infrastructure regarding this mechanism has not been fully elucidating [30,31]. A large cohort study conducted by Hsu et al. reported that the high LMR ratio was associated with better survival outcomes in gastric cancers patients, whereas there was no correlation between the given cut-off value and prognosis in our study [11]. In our study, LMR was similarly low in patients with poor prognoses, but no statistical difference was found. This may be due to the small number of patients included in our study. Our study may be helpful in raising questions about these values and shedding light on prospective randomized studies.

Platelets with more granules and secretory capacity are more easily triggered than platelets with fewer. Platelet size and platelet activity are thus related [11]. The present study is one of the few to investigate MPV in gastric cancer patients. We found that MPV was not satisfactorily predictive of prognosis. Previous research has suggested that MPV could be used as a biomarker for cancer diagnosis and follow-up [32,33]. In our reference study, it was emphasized that elevated postoperative MPV was an indicator of poor prognosis [34]. Different results may be due to the fact that the preoperative MPV was not predictive of the prognosis, whereas the elevated postoperative MPV might be a consequence of an inflammatory response [10].

The retrospective design was one of the limitations of our study. In the present study, we investigated the predictive prognostic values of different inflammatory markers in gastric cancer. We believe that prospective randomized or at least retrospective studies are needed to evaluate the relationship of inflammatory markers with postoperative changes and prognosis. In addition, the small number of patients in our center and the problems with follow-up records were other limitations of our study, therefore prospective studies are needed in this regard. Also, we aimed to test the cut-off values from the most-cited articles published in quality journals. Different results can be obtained with different cut-off values. Although most of the available studies report a positive relationship between these inflammatory markers and cancer prognosis, there is a limited number of articles that test this thesis and prove its accuracy. In our study, we used three cut-off values from the most-cited articles, which were observed to not affect the prognosis.

## Conclusions

Patients with a high preoperative CAR had a worse prognosis than those with a low preoperative CAR. Preoperative CAR can be used as a clinically accessible biomarker to assist clinicians in determining prognosis in patients with locally advanced gastric cancer. Prospective studies in several locations should be carried out to see if these low-cost, easily available, and simple inflammatory markers, which are intended for use in clinical practice, are truly beneficial.
